# Nintedanib in systemic sclerosis treatment: a case report

**DOI:** 10.1186/s13256-024-04433-2

**Published:** 2024-02-23

**Authors:** Maysoun Kudsi, Raghad Tarcha, Naram Khalayli

**Affiliations:** 1 Department of Rheumatology, Faculty of Medicine, Damascus University, Al-Mazzeh, Damascus, Syria; 2 Faculty of Medicine, Damascus University, Damascus, Syria

**Keywords:** Systemic sclerosis, ILD, Interstitial lung disease, Nintedanib, Forced vital capacity, SSc, Case report

## Abstract

**Background:**

Nintedanib was approved for the treatment of scleroderma and scleroderma-related interstitial lung disease, as it decrease the forced expiratory volume.

**Case presentation:**

A 48-year-old Asian female patient with systemic scleroderma 6 years ago developed breathlessness, nausea, heart palpation, and sudden severe occipital headache over the preceding week. She was receiving aspirin 81 mg/day and amlodipine 5 mg/day. Her diagnosis was diffuse scleroderma with pulmonary hypertension, interstitial lung involvement, and renal crisis. The modified Rodnan score was 18. We begin captopril at a dose of 12.5 mg, progressively escalating to 200 mg/day, and oral nintedanib was started at 150 mg. A total of 12 months after initiation of treatment, the patient’s kidney function was normal. The pulmonary function tests improved. The modified Rodnan score was reduced to 10. We did not encounter any side effects in our case due to nintedanib treatment.

**Conclusion:**

Treatment with nintedanib is crucial for slowing lung function decline. Diarrhea was the most common adverse event. Scleroderma renal crisis occurs in 10% of patients and typically presents with an abrupt onset of hypertension and kidney failure. The optimal antihypertensive agent for scleroderma renal crisis is an ACE inhibitor. The mainstay of therapy in scleroderma renal crisis has been shown to improve or stabilize renal function in approximately 70% of patients and improve survival in nearly 80% at 1 year. Nintedanib may be effective, and fairly safe to use. Further exploration is anticipated to advance a new period of systemic sclerosis treatment.

## Background

Systemic sclerosis (SSc) is a systemic connective tissue condition resulting in the activation of the immune system, leading to organ fibrosis, and vascular damage, with serious complications, and poor prognosis [1, 2]. Skin sclerosis degree correlates with poor prognosis in these patients [3]. Additionally, interstitial lung disease related to SSc, pulmonary hypertension and renal crisis is the most common cause of death in SSc [2]. A new era of fibrotic lesion therapies in SSc is highly needed [4]. In previous research, cyclophosphamide and autologous hematopoietic stem cell transplantation, which is used in specific and limited indications, improved cutaneous sclerosis, while methotrexate and mycophenolate mofetil did not show the same results, and their use in this was is off label [2, 5, 6]. The use of nintedanib, an oral tyrosine kinase inhibitor, in SSc and SSc-interstitial lung disease (ILD) treatment opened a new era of SSc treatment [7]. It can slow the rate of decrease in pulmonary function in adult patients with this disease [8].

Vascular endothelial growth factor (VEGF), transforming growth factor (TGF)-β, MAPK, PI3K/AKT, JAK/STAT and WNT/β-catenin signalling are the molecular pathways modulated by nintedanib, and these pathways are controlled by intercellular adherence junctions, and by central carbon metabolism. It affects the restriction of neo-angiogenesis by inhibiting several growth factors [9].

It inhibits the profibrotic mediators such as transforming growth factor-β, fibroblast growth factor and platelet-derived growth factor, thus reducing fibroblast activity. The overexpression of FAK can reverse the nintedanib inhibitory effect on pulmonary fibrosis [10].

## Case presentation

A 48-year-old Asian female patient with known systemic scleroderma 6 years ago, diagnosed according to the American College of Rheumatology Criteria [11], which in our patient are telangiectasia over her face, skin thickening of both hands accompanied by pain, and ulcers in the digits, presented to the hospital with complaints of cough, dyspnea and orthopnea. Over the preceding week, she developed breathlessness, and 24 hours previous, she suffered from nausea, heart palpation and sudden severe occipital headache which did not respond to paracetamol.

No previous medical history, family history and psychosocial history was found. She was receiving aspirin 81 mg/day and amlodipine 5 mg/day. The patient was started on 20 mg of methotrexate weekly for 4 years, but she developed nausea, vomiting and abdominal pain, so methotrexate was discontinued. During the last 2 years, she could not start treatment with tocilizumab 162 mg once weekly subcutaneously due to allergic reaction.

Her vital signs on admission were a respiratory rate of 20 breaths/minute, a heart rate of 106 beats/minute, blood pressure of 224/130 mm Hg and body temperature of 37.4 °C. The patient was oriented but fidgety, and she appeared to be short of breath.

On inspection, findings were loss of wrinkling, and telangiectasia over the face and diffuse skin thickening of both hand and foot. She had soft crackles in both lungs by auscultation, and diffuse abdominal tenderness. Other systemic examination findings were unremarkable.

The laboratory evaluation showed white blood cells at 6.3 K/microL (normal: 4.0–11.0) with 69.5% of neutrophils (normal: 50%–70%). Haemoglobin was 11.3 g/dL (normal: 12–16), platelet count 155,000 K/uL (normal = 150,000–400,000), procalcitonin < 0.5 ng/mL, albumin 4.2 g/dL (normal: 3.5–5.5), alanine transferase (ALT) 23 U/L (normal: 7–55), aspartate transferase (AST) 38 U/L (normal: 8–48), lactate dehydrogenase (LDH) 1398 U/L (normal: 14–280 in adults), erythrocyte sedimentation rate (ESR) 24 mm/hour (normal: 0–20) and C-reactive protein (CRP) 3.2 mg/L (normal: < 6) and creatinine 2.5 mg/dL (normal > 1.2). Hematuria and proteinuria were found in urinalysis. The 24-hour protein analysis was 231 mg/24 hours (normal: < 150).

The immune profiles – rheumatoid factor, anti-cyclic citrullinated peptide antibody, anti-La, anti-Ro, perinuclear antineutrophil cytoplasmic antibody and antineutrophil cytoplasmic antibody – were negative. The antinuclear antibody (ANA) was positive at 1/160, with a speckled pattern, and antitopoisomerase 1 anti-Scl70 was positive by ELISA. Complements were within normal limits.

The pulmonary function tests showed a reduced diffusion capacity for carbon monoxide (DLCO; 58%) and decreased forced vital capacity (FVC; 68%), but the ratio of forced expiratory volume in 1 second/forced vital capacity (FEV1/FVC; 92%) was normal according to spirometry.

Electrocardiogram (ECG) revealed tachycardia, and the echocardiography showed pulmonary hypertension at 40 with ventricular dilation accompanied by tricuspid regurgitation.

**Fig. 1:**
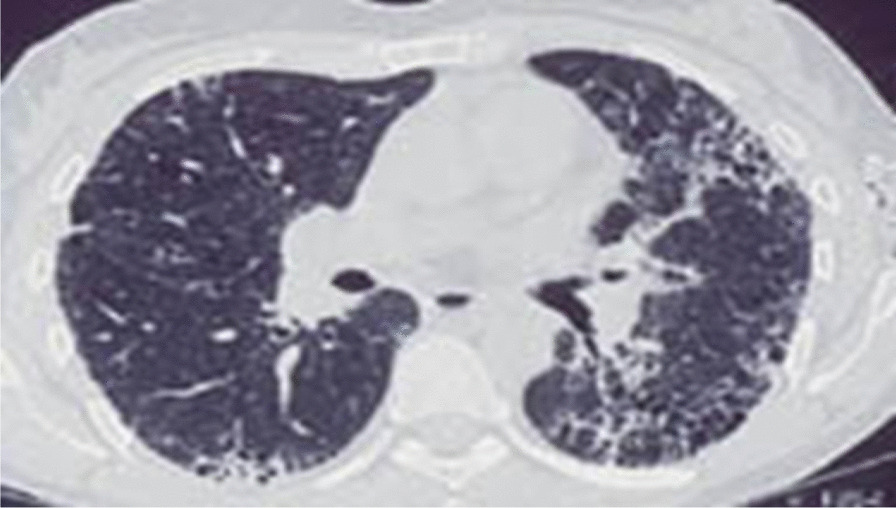
Chest high-resolution computed tomography

Chest X-ray showed reticular opacities, and chest high-resolution computed tomography (HRCT) revealed findings compatible with the interstitial pneumonia pattern, as it showed bilateral and symmetrical ground-glass opacification and smooth thickening of the interlobular septa (Fig. 1).

According to the above findings, the diagnosis was diffuse scleroderma with pulmonary hypertension and interstitial lung involvement, and the renal crisis was done. The modified Rodnan score was 18.

We begin captopril at a dose of 12.5 mg. We progressively escalated the dose in 25 mg increments at 8-hour intervals to 200 mg/day divided into three daily doses, which controlled the blood pressure within 72 hours, and the value of creatinine stabilized to 1.6 mg/dL.

Oral nintedanib was started at 150 mg twice daily. After 15 days the patient was discharged on 200 mg/day captopril divided into three daily doses and nintedanib 150 mg twice daily.

The laboratory tests on discharge were creatinine 1.4 mg/dL (normal > 1.2 mg/dL), and there was no hematuria and proteinuria in the urinalysis. The 24-hour protein analysis was 178 mg/24 hours.

A total of 6 months after initiation of treatment, the patient’s kidney function recovered completely. The pulmonary function tests improved, showing DLCO (62%), FVC (72%) and FEV1/FVC ratio (92%) as normal. The modified Rodnan score was also reduced to 11. Captopril was decreased to 150 mg/day divided into three daily doses and nintedanib to 100 mg twice daily.

A total of 12 months after initiation of treatment, the patient’s kidney function was normal. The pulmonary function tests improved more, showing DLCO (68%), FVC (79%), and FEV1/FVC ratio (92%) as normal. The modified Rodnan score was also reduced to 10. We did not encounter any side effects in our case due to nintedanib treatment.

Our study has been reported in line with the SCARE Criteria 2020 [12].

We have registered our study in Researchregistry.com – for all human studies – charge; the unique identifying number is 9333 [13].

## Discussion

Treatment of scleroderma includes non-steroidal anti-inflammatory drugs, a low dose of corticosteroids, immunosuppressants and biologics. Biologics treatment includes anti-CD20, TNF ALPH inhibitors and anti-fibrotic treatments [2, 14].

Serum levels of TNF-α are elevated in patients with SSc and favour the development of pulmonary fibrosis and pulmonary arterial hypertension. Inflammatory arthritis can occur in patients with SSc. Infliximab and etanercept may improve inflammatory arthritis and disability in SSc. TNF-α inhibitors reduce the systemic inflammation and improve the endothelial function, decreasing the risk of pulmonary arterial hypertension progression and of acute cardiovascular and/or cerebrovascular events [14].

Treatment with nintedanib is crucial for slowing lung function decline, improving clinical outcomes and reducing the risk of acute exacerbations by minimizing the annual rate of decline in forced vital capacity in randomized controlled trials (RCT) [10]. Furthermore, efficacy was observed in patients with ILD with a progressive fibrosing phenotype, including those with SSc and other connective tissue diseases [15, 16].

Data suggest that different interstitial lung diseases with a progressive pulmonary fibrosis phenotype, including SSc-related ILD [17], can share similar pathogenetic and biological pathways and could be amenable to anti-fibrotic therapies. Indeed, historical management strategies have failed to identify potential treatments once progression has occurred despite available drugs [15, 18].

In the SENSCIS trial in patients with systemic sclerosis-associated interstitial lung disease (SSc-ILD), nintedanib reduced the rate of decline in forced vital capacity (FVC) versus placebo, with adverse events that were manageable for most patients. An open-label extension trial, SENSCIS-ON, is assessing safety and FVC decline during longer-term nintedanib treatment [19]. It takes approximately 6 months of therapy with nintedanib before an improvement in lung volumes is seen [18, 19].

In patients with SSc-ILD, safety profiles observed during the trial were consistent with those observed in patients with idiopathic pulmonary fibrosis. Diarrhoea was the most common adverse event, leading to discontinuation or dose reduction of nintedanib [15, 16, 18]. No adverse event was noticed in our case.

We demonstrate that nintedanib effectively inhibits the endogenous as well as cytokine-induced activation of SSc fibroblasts and exerts potent antifibrotic effects in different complementary mouse models of SSc. These data have direct translational implications for clinical trials with nintedanib in SSc [17, 18].

Nintedanib comes as a capsule to take by mouth with a lot of water. It is usually taken with food every 12 hours (twice a day), and it should be taken at around the same times every day [18, 19].

Kidney disease in SSc is common. Scleroderma renal crisis (SRC), which occurs in 10% of patients, is the most serious complication. Kidney involvement is more frequently observed in the context of extensive diffuse skin disease and typically occurs in the first 2 years. SRC typically presents with an abrupt onset of moderate to marked hypertension and kidney failure without signs of glomerulonephritis [9].

Our patient had a sudden onset of marked hypertension and kidney failure with signs of glomerulonephritis, as hematuria and proteinuria were found in urinalysis and 24-hour protein analysis.

Several risk factors for SRC have been identified, including the presence of diffuse skin involvement, the presence or absence of some serum autoantibodies and the use of certain drugs, such as glucocorticoids and cyclosporine [9, 20]. There was no risk factor in our case.

Thrombotic thrombocytopenic purpura (TTP), anti-neutrophil cytoplasmic antibody (ANCA)-associated glomerulonephritis, crescentic rapidly progressive glomerulonephritis (RPGN) and atypical hemolytic uremic syndrome (aHUS), which remain uncommon presentations of acute renal failure in SSc, can present similarly to SRC [9, 20].

The principal goal is to return the patient to their previous baseline blood pressure within 72 hours. The optimal antihypertensive agent for SRC is an ACE inhibitor (ACEi), which should be introduced or the dose increased. A short-acting ACEi (for example, captopril), as we used, may theoretically be preferable in the hemodynamically unstable patient. Other blood pressure (BP)-lowering agents such as angiotensin receptor blockers (ARBs) may be used. Despite appropriate ACEi therapy, dialysis is needed in approximately 60% of patients with SRC [20]. Our patient did not need dialysis.

SRC can progress to end-stage kidney disease (ESKD) for 1–2 months, with death usually occurring within 1 year [4]. The mainstay of therapy in SRC is effective and prompt blood pressure control, which has been shown to improve or stabilize renal function in approximately 70% of patients and improving survival in nearly 80% at 1 year [9, 20]. The renal function of our patient had returned to normal.

We did not find any data about using nintedanib in the treatment of SRC. The improvement in SRC in our patient may be due to the use of ACE inhibitors.

## Conclusion

Nintedanib may be effective, and fairly safe to use in systemic sclerosis cases, even in the presence of a renal crisis. How to use this medicine else remains to be determined grounded in real-world data. Further exploration is anticipated to advance a new period of SSc treatment. As the clinical course of SSc-ILD is variable, further research should aim at identifying clinical, biological and imaging biomarkers for predicting the progressive fibrosing phenotype and at establishing an evidence-based treatment algorithm.

## Data Availability

Not applicable.

## Abbreviations

aHUS: Atypical hemolytic uremic syndrome
ALT: Alanine transferase
ANA: Antinuclear antibody
ARBs: Angiotensin receptor blockers
AST: Aspartate transferase
CRP: C-reactive protein
DLCO: Diffusion capacity for carbon monoxide
ECG: Electrocardiogram
ESKD: End-stage kidney disease
ESR: Erythrocyte sedimentation rate
FVC: Forced vital capacity
HRCT: Chest high-resolution computed tomography
ILD: Interstitial lung disease
LDH: Lactate dehydrogenase
RPGN: Rapidly progressive glomerulonephritis
SRC: Scleroderma renal crisis
SSc: Systemic sclerosis
TTP: Thrombotic thrombocytopenic purpura
